# The Smarter Safer Homes Solution to Support Older People Living in Their Own Homes Through Enhanced Care Models: Protocol for a Stratified Randomized Controlled Trial

**DOI:** 10.2196/31970

**Published:** 2022-01-24

**Authors:** Qing Zhang, Marlien Varnfield, Liesel Higgins, Vanessa Smallbon, Julia Bomke, John O'Dwyer, Joshua M Byrnes, Melissa Sum, Jennifer Hewitt, Wei Lu, Mohanraj Karunanithi

**Affiliations:** 1 Australian eHealth Research Centre Commonwealth Scientific and Industrial Research Organisation Herston Australia; 2 Centre for Applied Health Economics Griffith University Brisbane Australia; 3 Anglicare Southern Queensland Brisbane Australia; 4 Integratedliving Australia Newcastle Australia

**Keywords:** smart home, aged care, objective activity of daily living, randomized trial, wireless sensor network, older adults, care, methodology, platform, benefit, utilization, support, self-management, digital health

## Abstract

**Background:**

An aging population, accompanied by the prevalence of age-related diseases, presents a significant burden to health systems. This is exacerbated by an increasing shortage of aged care staff due to the existing workforce entering their retirement and fewer young people being attracted to work in aged care. In line with consumer preferences and potential cost-efficiencies, government and aged care providers are increasingly seeking options to move care and support to the community or home as opposed to residential care facilities. However, compared to residential care, home environments may provide limited opportunity for monitoring patients’ progression/decline in functioning and therefore limited opportunity to provide timely intervention. To address this, the Smarter Safer Homes (SSH) platform was designed to enable self-monitoring and/or management, and to provide aged care providers with support to deliver their services. The platform uses open Internet of Things communication protocols to easily incorporate commercially available sensors into the system.

**Objective:**

Our research aims to detail the benefits of utilizing the SSH platform as a service in its own right as well as a complementary service to more traditional/historical service offerings in aged care. This work is anticipated to validate the capacity and benefits of the SSH platform to enable older people to self-manage and aged care service providers to support their clients to live functionally and independently in their own homes for as long as possible.

**Methods:**

This study was designed as a single-blinded, stratified, 12-month randomized controlled trial with participants recruited from three aged care providers in Queensland, Australia. The study aimed to recruit 200 people, including 145 people from metropolitan areas and 55 from regional areas. Participants were randomized to the intervention group (having the SSH platform installed in their homes to assist age care service providers in monitoring and providing timely support) and the control group (receiving their usual aged care services from providers). Data on community care, health and social-related quality of life, health service utilization, caregiver burden, and user experience of both groups were collected at the start, middle (6 months), and end of the trial (12 months).

**Results:**

The trial recruited its first participant in April 2019 and data collection of the last participant was completed in November 2020. The trial eventually recruited 195 participants, with 98 participants allocated to the intervention group and 97 participants allocated to the control group. The study also received participants’ health service data from government data resources in June 2021.

**Conclusions:**

A crisis is looming to support the aging population. Digital solutions such as the SSH platform have the potential to address this crisis and support aged care in the home and community. The outcomes of this study could improve and support the delivery of aged care services and provide better quality of life to older Australians in various geographical locations.

**Trial Registration:**

Australian New Zealand Clinical Trials Registry ACTRN12618000829213; https://tinyurl.com/2n6a75em

**International Registered Report Identifier (IRRID):**

DERR1-10.2196/31970

## Introduction

### Background and Rationale

Globally, the population is aging. It is expected that by 2050, 1 in 6 people will be over the age of 65 years, representing a significant increase from the current rate of 1 in 11 [1]. Australia’s aged population is steadily increasing. A fertility boom from 1946 to 1965, coupled with advances in health care and associated increased life expectancy, resulted in the proportion of the population aged 65 years and over to increase from 11.1% to 14.24% between 2000 and 2020 [2], and it is expected to reach 25% by 2056 [3]. Currently, there are 5 people of working age for every person over 65 years old; however, this number is expected to drop to 2.7 by 2050 [4]. The dwindling of the workforce is further exacerbated in aged care due to the predominance of older workers (48-50 years) [5] reaching retirement and fewer young workers being attracted to working in this area.

With a large percentage of our aging population facing injury, disability or chronic disease, and requiring regular medical care, health expenditure is rising faster than economic growth [6]. Several reports demonstrate the negative impact of an aging population on health care expenditure [7-9]. These reports indicate that the cost of health care increases with age, doubling between 45 and 65 years, and doubling again between 65 and 85 years. The majority of the health care spent on aging is attributed to public hospital funding, with a 5-fold increase on those aged 75 to 84 years and over compared to the median per capita amount [10]. Furthermore, the proportion of those accessing residential aged care facilities increases significantly for those aged 80 years and over.

In line with consumer preferences and potential cost-efficiencies, government and aged care providers are increasingly seeking options to move care and support to the community or home. In Australia, the provision of home-based care has previously been shown to not only significantly cost less (AUD 6.7 billion=US $4.9 billion) compared to residential aged care (AUD 13.6 billion=US $9.9 billion) [11] but also reduced the number of required residential aged care placements. However, this may also put further pressure on families to meet the health, social, safety, and other daily needs of their older members, whereas many families have limited time, physical, or financial capacity to attend to their aging relative’s everyday needs and care.

The use of assistive technologies has the potential to support home-based care for older Australians in the community, deliver reduced savings in health expenditure, and increase functional independence [12]. For example, it has been estimated that avoiding as few as 10% of falls would reduce hospital costs by AUD 85 million (US $61.8 million) [13]. Regular medical care, increasing awareness of health and of healthy lifestyles, and monitoring of daily activities from assistive technologies have the potential to further reduce hospital costs through avoidance or early detection of degeneration in health and well-being.

To address issues of limited family support, residential care placement availability, and the impending shortage of the aged care workforce and associated health and aged care costs, we developed a smart home platform called “Smarter Safer Homes” (SSH), which features a lifestyle-based approach in the design and implementation to enable older people to live longer in their own homes, with their choice of how they interact with the technology and engage family and/or aged care support. The SSH platform integrates wireless home sensor and health monitoring devices to allow engagement of informal (eg, family) support and formal aged care services. One of the novel features of the platform is the analytics that capture an individual’s profile of activities of daily living (ADL) from which personal-level functional independence or ability can be determined. 

The “smart home” concept was introduced in the 1980s when it was used to support independent living and older peoples’ health and aging [14]. Along with the emergence of new technology in mobile computing, smart sensors, and the Internet of Things (IoT), the smart home has become topical and relevant with respect to in-home automation and assistance for health and well-being [15-17]. Although various technologies used in smart homes, such as wireless sensor networks [18] and activity recognition algorithms [19], have been evaluated individually, very few randomized controlled trials (RCTs) have been performed to evaluate the entire smart home platform. This includes whether or not implementation of smart home technologies is possible in everyday homes [20], or if the smart home can impact certain clinical diseases such as cognitive decline [21,22] or risks of falls [23].

To evaluate our smart home platform for its use in supporting aged care for residents living at home, an RCT was undertaken. The aim of the study was to evaluate the SSH platform to support remote care delivery and management of home-based aged care. To our knowledge, our trial is the first RCT to evaluate changes in the health and well-being of older adults living independently using clinically validated instruments. It is also the first to conduct a health care cost-utility analysis to compare the cost-effectiveness of a smart home intervention.

### Objectives

The key research question of this trial is whether implementing smart home technology–enabled self-management and care delivery can maintain or improve the impact of care provided by aged care service providers to older people living independently in their own homes.

The aims of the trial were to evaluate the impact of implementing an innovative home care service delivery model via technology on: (1) the impact of care provided by aged care service providers in response to needs arising from physical or sensory impairments for older people living independently in their homes; (2) quality of life for older people living independently in their own homes; (3) factors associated with ADL and instrumental activities of daily living (iADL); (4) depression in older people living independently in their own homes; (5) health service utilization, including presentation to hospital (admitted and emergency department), attendances to general practitioners, and other Medicare-funded community health services; (6) existing model of care, service design, adoption, and aged care service provider experiences; (7) carer burden (informal carers); and (8) costs to the government as a result of deployment of the SSH platform.

## Methods

### Trial Design

The trial was designed as a single-blind, stratified RTC. Participants were divided into two groups with an allocation ratio of 1:1. Trial participants could not be blinded as the intervention required physical installation of equipment in their homes. Researchers undertaking data analyses are blinded to which participants received the intervention. Access to community health services has previously been identified to vary by geographic location [24]. To ensure balance between the intervention and control groups relative to this potential confounder, stratification based on region (ie, metropolitan or regional area) is used.

### Study Setting

#### Overview

Participants living in community independent dwellings were recruited from two geographic areas, metropolitan and regional, based on the Rural Remote Area and Metropolitan Area classification criteria [25]. Three participating aged care service providers deliver aged care services to one or both geographical areas. Specifically, Anglicare Southern Queensland recruited participants from metropolitan and regional areas in Queensland (QLD), integratedLiving Australia recruited participants from metropolitan and regional areas in QLD, and All About Living is recruited participants from metropolitan areas in QLD.

#### Anglicare

Anglicare Southern Queensland is a member of the Anglicare Australia Network providing support to aged Australians in partnership with government and other support organizations in response to identified care needs throughout southeast QLD. They offer a range of specialist services within indigenous, homeless, multicultural, and rural and remote communities. The workforce of Anglicare Southern Queensland stretches from Cairns to Coolangatta, and from Birdsville to Brisbane. Recruitment included participants from metropolitan and regional areas in QLD.

#### integratedLiving Australia

integratedLiving Australia receives funding from the Australian and state governments to provide care services. They provide in-home support services to older people in regional, rural, and remote Australia (including northern and eastern QLD) and have been focusing on ensuring equitable access for health support to these communities. Recruitment included participants from regional areas in QLD.

#### All About Living

All About Living partners with federal, state, and local government departments, along with several community organizations to deliver a range of high-quality services. All About Living has developed governance, management, and service delivery expertise and excellence that enables consistent service delivery. Recruitment included participants from metropolitan areas in QLD.

### Participants

It was anticipated that the trial would include 200 individuals (see the Sample Size subsection below) aged 65 years and older. Table 1 provides a breakdown of the number of individuals planned to be recruited from each service provider in the two areas.

**Table 1:** Number of participants planned to be recruited.

Area	Anglicare, n	integratedLiving, n	All About Living, n
Metropolitan	100	25	20
Regional	40	15	0

### Collaborations With Aged Care Partners

One of the unique features of this RCT is the collaboration between the researchers and the aged care service providers. The relationship was established as a true partnership with a representative of each aged care service provider attending monthly project meetings, the risk and safety committee meeting, and regularly being consulted with as the experts on their own participants. Additionally, while asked to record certain data points, the aged care service providers were asked to develop their own workflows in how they respond to trends produced by the SSH platform and how they check on their participants if flags are raised by the data from the SSH platform. Aged care service providers reported any concerns about data (eg, system glitches) to the project manager and these were reported to the engineering team immediately. The aged care service providers are also involved in all reviews of reports, case studies, and documents associated with the project. A true teamwork collaboration was formed between the researchers and the aged care service providers, which is key to the success of the research as a whole.

### Collaborations With Consumers

In addition to the aged care service providers, the project team was fortunate to have the input of a consumer representative (ie, a person with a lived experience in this area of aging). The consumer representative also attended monthly project meetings and was able to provide guidance on how participant information should be written and delivered, how professionals should approach research participants, and provide the lived experience viewpoint to the research. Unfortunately, the consumer representative ceased attending meetings when the COVID-19 lockdowns commenced in March 2020. The team recognizes the contribution of the consumer representative during the time they were involved and how their influence enhanced the development, design, and implementation of the recruitment phase in particular.

### Eligibility Criteria

The inclusion criteria for this trial were people aged 65 years and older; living at home, in the care of a designated aged care service provider; and English-speaking with proficiency in written English. The exclusion criteria were people residing in long-term residential care, not able to give informed consent due to reasons such as severe cognitive impairment, not willing to leave their electricity on overnight, and people residing with others.

### Intervention

**Figure 1:**
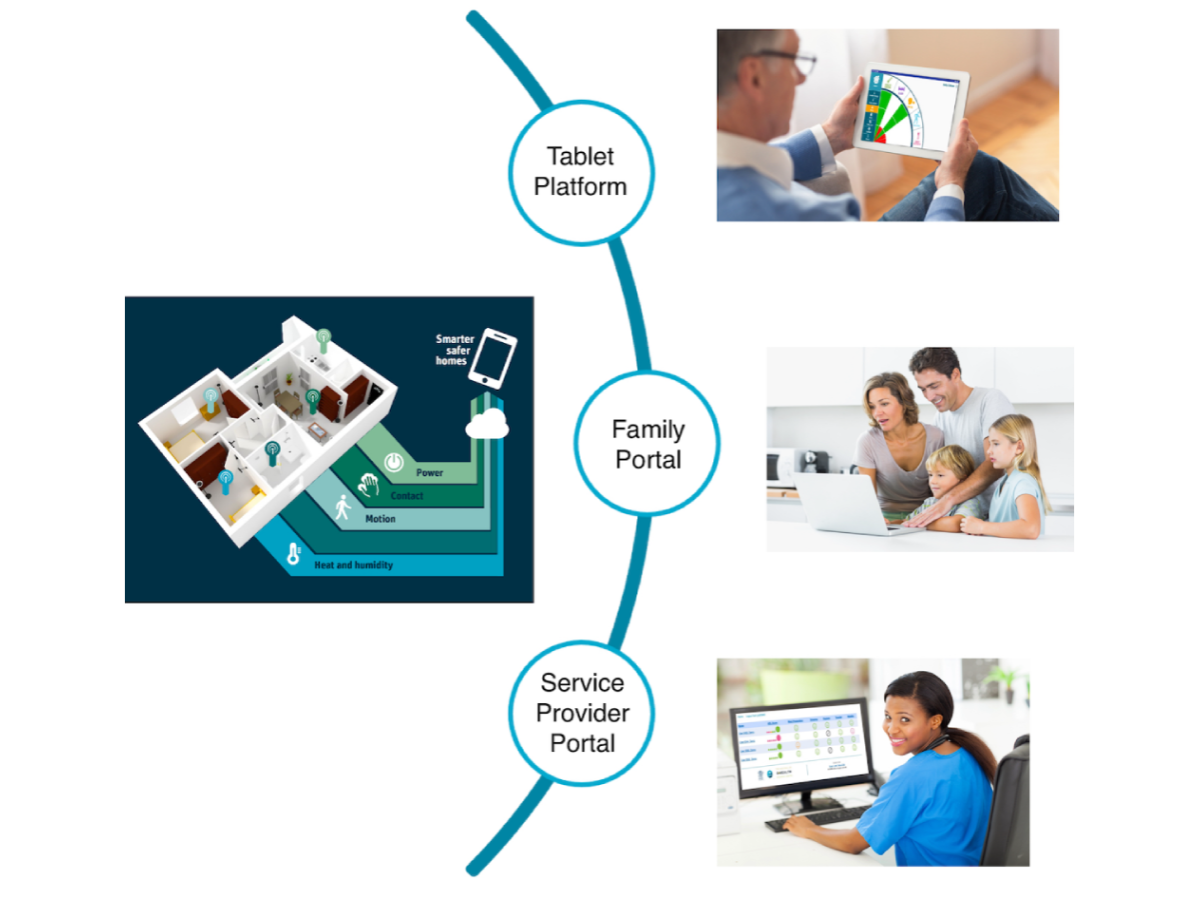
Overview of the Smarter Safer Homes platform.

The intervention involved use of the SSH platform to assist aged care service providers to monitor and provide timely support to their clients. As shown in Figure 1, the SSH platform comprises a client module (data presentation) with a tablet app, family portal, and service provider portal.

The intervention also provided participants the choice to view their data/progress on the tablet app. This intervention was not a real-time solution, with all data viewed in the platform being from the previous 24-hour period. Participants and aged care service providers were made aware of this multiple times in multiple ways, as there was a risk they would rely on the system as an alert if an adverse incident occurred in the home, such as a fall. All usual care was maintained for all participants, regardless of their allocation to the research groups. Therefore, for those in the intervention group, the SSH installation was an added layer of care intervention.

**Figure 2:**
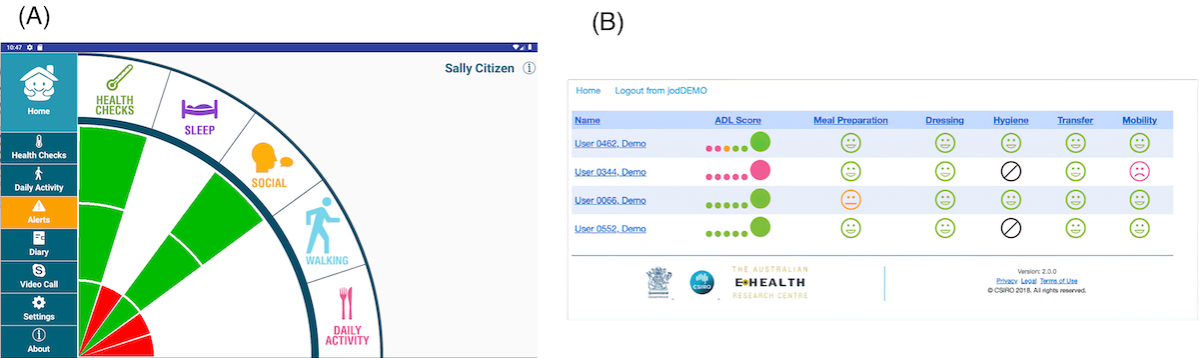
Screenshots of (A) the Smarter Safer Homes (SSH) app, and (B) family and service provider portal interfaces.

The interface of the app was co-designed with a network of similar cohorts during the inception of the SSH platform [26]. An example of the SSH app dashboard, as shown in Figure 2A, reflects the daily status of health and well-being, indicated by different sized and colored rays. A full-length green ray indicates the status within the normal range. An amber ray with two-thirds of the length raises concerns about the status, while a red ray with one-third of the length warns that the health and well-being status is abnormal and may require timely intervention from carers. The family portal has the same interface design, but with limited functionality to keep significant others informed about the well-being of their loved one (the participant). Figure 2B shows the interface design of the service provider portal, which provides access to a formal carer (eg, the participant’s aged care service provider) to monitor the participant’s condition and ADLs. The color schema used in this portal is the same as that used in the SSH app (ie, green dots and grinning faces indicate normal status, amber dots and neutral faces indicate concerned status, while red dots and frowning faces indicate abnormal status). Interventions from aged care service providers, such as phone calls, will be initiated if red dots observed.

The features of the SSH platform include a sensor-based in-home monitoring system (data collection), a cloud computing server (data analyses), and novel analytics to determine functional independence. The novel measure of functional independence features the provision of an objective and personalized measure of ADL components and scoring through nonwearable and nonintrusive sensors in the home environment, and the ability to correlate this measure with self- or care-reported status of health and well-being. The domains (mobility, transfer, hygiene, and meals) of the ADL score are derived through aggregation and artificial intelligence analytics from a range of sensors deployed in the home (eg, motion, accelerometers, power, and the temperature/humidity within a room). Individualized functional independence (Objective ADL [O-ADL]) is measured using the same framework as the ADL assessment performed in clinical settings. This O-ADL is not only an objective clinical assessment using home environmental sensors but is further personalized through learning an individual’s activity and profile relative to their health and functional status. This then references their ongoing functional status over time, enabling timely intervention.

### Outcomes

#### Baseline Characteristics

Baseline characteristics were collected to facilitate the identification of systematic differences between study groups that may increase the risk of bias.

#### Primary Outcome

The primary outcome was the Australian Community Care Outcome Measurement tool (ACCOM) [27].

#### Secondary Outcomes

The secondary outcomes included change in health-related quality of Life, as measured by the Five-Dimension EuroQuol (EQ5D 5-L) questionnaire [28]; Katz ADL [29] and instrumental ADL (Lawton iADL [30]); depression (Geriatric Depression Scale [31]); health service utilization, including community prescription medicines (collected based on Pharmaceutical Benefit Scheme [PBS] claims data), community health services (including nonreferred medical attendances such as general practitioner appointments, attendances with medical specialists, pathology, diagnostic imaging, and Allied Health attendances collected from Medical Benefit Scheme [MBS] claims data), hospital attendances (same day and overnight admitted), and emergency department presentations (collected from QLD public hospital data); changes in service design, adoption, and aged care service provider experiences (based on provider staff interviews); care giver burden (Zarit Burden Interview [ZBI]-12 [32]); and costs to deliver the SSH supported care.

### Participant Timeline

The trial recruited its first participant in April 2019 and data collection of the last participant was completed in November 2020**.** The study received participants’ health service utilization data in June 2021.

### Sample Size

**Figure 3:**
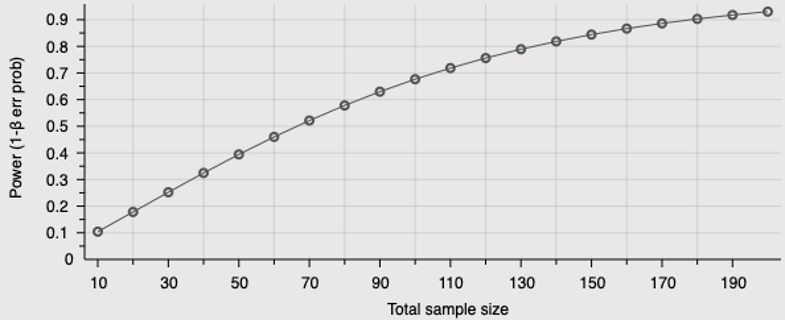
Total sample size and statistical test power.

The primary outcome of this trial, the impact of community care for older people, will be evaluated using the ACCOM tool [27], a set of measures of community care suitable for use in the Australian context. The ACCOM mainly uses questions from the Adult Social Care Outcome Toolkit (ASCOT) [33], which is a validated measure of social care related to quality of life. Power was based on a randomized trial design with a clinically important difference of 10% on the primary outcome of ASCOT with a mean 0.80 (SD 0.16) distribution (ie, 0.08, based on previous research [33]). With the threshold of rejecting the null hypothesis of *α*≥.05, effect size of 0.5 (0.08/0.16), and the same allocation ratio of the two groups in our trial. Figure 3 illustrates the relations between total sample size and statistical power.

To achieve 80% statistical power, we needed a total of 134 participants. Allowing for a 30% attrition rate, the sample size of this trial was computed to be 200 participants. We did not anticipate an attrition rate greater than 30% as all participants were existing clients of the participating aged care service providers and received regular visits throughout the trial period. The intervention and outcome measurement collection processes were designed to minimize participant burden. In addition, the power analysis did not consider the repeated-measures design of the trial. Repeated measures increase the power of the trial to find a significant result and appropriate statistical techniques will be applied to ensure that all data available are used in the analyses.

### Recruitment

Three project officers were employed to recruit and consent participants for the trial. The project officers made contact with potential participants, after introduction by the aged care service provider. The project officer, via phone call, briefed the individual on the trial, assessed their interest in participating, and checked their eligibility. If the individual agreed to participate, they were asked to nominate their witness, who could be the informal carer (which could include family members or another person that they hold a close relationship with such as a friend or neighbor) to be included in a future face-to-face meeting. During the face-to-face meeting, the project officer confirmed the internet connectivity at home and the cognitive awareness of potential participants. At this point, cognition levels were assessed according to the participant’s ability to demonstrate understanding of what the project officer was saying and how the potential participant responded to questions. It was identified during the planning of the trial that research participants would incur a small out-of-pocket fee for the use of their own electricity and internet data. This was calculated to be less than AUD 5 (US $3.6) per month. The budget allowance was established to ensure that all individual participants were repaid for this out-of-pocket expense after their contribution to the research was complete. For example, a participant received an AUD 60 (US $43.20) gift card for 12 months of participation (5×12 months=60).

### Assignment of Intervention

#### Allocation

Stratified randomization was used with the strata defined by geographical areas (ie, metropolitan and regional). Within each area, simple randomization (ie, randomization based on a single sequence of random assignments [34]) was performed and participants were assigned to either the provider’s usual care or their services designed and delivered through the SSH technology platform (smart home group). Randomization of participants was undertaken using a computer program and carried out by an independent researcher using anonymized identifiers. The random allocation sequence was delivered through the REDCap application. Allocation occurred after the baseline survey had been completed.

#### SSH Kit Installation

Once participants had been consented, a set of surveys was administered by the project officer. The participants allocated to the intervention group received an SSH kit. Installation of the SSH kit (tablet, hub, sensors) was coordinated by the project officer, typically within 2 weeks of consent and randomization. A user sheet that describes how to interact with the tablet and interpret the information presented on the tablet SSH app was included with the kit. The project officer also demonstrated to the participant how to use the tablet and the SSH app during installation. To obtain valid baseline data after installation, the SSH system gathered 14 days of the participants’ normal living routines in their homes. After the baseline data had been gathered, the identified aged care service providers and the nominated family portal users were notified of their user ID, password, and access instructions. During the trial period, the project officer was the point of contact for any problems or questions. Technical issues that were not easily addressable were escalated to the research technical team who worked to find efficient and effective solutions. All reported issues, no matter how minor, were reported to the project manager for inclusion in the monthly project reports. If a participant wished to withdraw from the trial, the project officer arranged for an uninstall to occur as quickly as possible from the home. Upon completion of the project, all homes had their trial equipment removed by the project officer. Participants allocated to the usual care group continued to receive their existing care and social services in line with local aged care service provider protocols for the 12 months of the trial. They did not have any SSH equipment installed in their homes.

### Measurements and Data Collection

#### Overview

Data were collected from participants, informal carers, and aged care service providers from state and national government data sources and home-based sensor systems. These included surveys, raw sensor data, and interview information. The state and national government data sources included hospital linkage data and MBS/PBS data. The measure, context, score meaning, and time points of collection are presented below.

#### Survey Questionnaires

##### Baseline

The project officers administered a baseline survey to all consented participants. Questionnaires included in the baseline survey are listed in Table 2. Note that the Abbreviated Mental Test Score (AMTS) was administered by a project officer prior to administering the baseline survey. Follow-up questionnaires at the mid-trial point (around 6 months) and end-trial point (around 12 months) included the same battery of questionnaires except for the demographics and AMTS. The baseline survey was delivered to participants during the consent face-to-face meeting and took approximately 45 minutes to complete.

**Table 2:** Survey questionnaires.

Domain	Measure	Context	Score meaning/presentation	Time point		
				Start	Mid	End
Demographic information	Individual questions	Gender, age, weight and height (BMI), occupation, marital status, income, computer skills, social media, and NBN^a^ connectivity	Individual and coded scores	✓		
Cognitive level	Abbreviated Mental Test Score (AMTS) [35]	To establish baseline cognition (10 questions)	Maximum score 10; a score of less than 7 or 8 suggests cognitive impairment	✓		
Impact of care: participant	ACCOM^b^ measure (adapted from ASCOT^c^ for the Australian population) [33]	Self-completion (eight attributes): control over daily life, personal cleanliness and comfort, food and drink, personal safety, social participation and involvement, occupation, accommodation cleanliness and comfort, dignity. Additional questions: subjective rating of health, open question	Four levels: Ideal State, No needs, Some needs, High needs; ASCOT scoring [36]	✓	✓	✓
Health-related quality of life	EQ5D^d^ [37]	Five generic questions on health status: mobility, self-care, usual activities, pain/discomfort, anxiety/depression; respondents’ self-rated health is recorded on a vertical, visual analog scale (VAS: 0-100)	Results presented as an index value (Australian Scoring algorithm); VAS presented as a number from 0 to 100, with 0 indicating the worst and 100 indicating the best imaginable health state	✓	✓	✓
Activities of Daily Living (ADL)	Katz’s ADL [29]	Assesses basic ADLs: feeding, continence, transferring, toileting, dressing, bathing	Maximum score of 6 points indicating fully independent, 4 points indicating moderately impaired, and 2 points indicating severely impaired	✓	✓	✓
Instrumental Activities of Daily Living (iADL)	Lawton’s iADL [30]	Assesses a person’s ability to perform daily tasks, measuring eight domains: using the telephone, shopping, preparing food, housekeeping, doing laundry, using transportation, handling medications, handling finances	Summary score from 0 (low function) to 8 (high function)	✓	✓	✓
Depression	Geriatric Depression Scale (Short Form) [31]	15-item version, used to identify depression in older people	Scores >5 (yes) suggest presence of depression; scores >10 are almost always depression	✓	✓	✓

^a^NBN: National Broadband Network.

^b^ACCOM: Australian Community Care Outcome Measurement.

^c^ASCOT: Adult Social Care Outcome Toolkit.

^d^EQ5D: 5-dimension EuroQuol.

##### Follow-Up

Participants were contacted by a project officer to complete the same surveys at mid-trial and end-trial. These were preferably conducted over the phone, and particularly during imposed COVID-19 safety measures. For the intervention group, an uninstall of the SSH kit typically took place at the same time as the end-trial survey was administered. Follow-up was systematized through the REDCap system. For participants who withdrew from the trial, all data collected up to the point of withdrawal are included in the analysis of the study, unless formally requested not to be by the participant. This was outlined in the Participant Information and Consent Form (Multimedia Appendix 1) and participants were asked to consent to the inclusion of their data.

#### Sensor Data

All in-home raw sensor data were transferred to the IoT router and then collected directly to a secured server where all sensor and participant data were gathered. Storage of and access to the project data within the data center were governed by privacy policy and procedures, and limited to the investigators on this project. Data center operations staff had access to the data to perform their normal duties (eg, database backup).

The data gathered through the in-home sensors were categorized into daily living activity domains, as listed in Table 3.

The data were not live-monitored in the trial. Participants were made aware of this prior to the trial commencing to ensure that a false sense of security was not assumed.

**Figure 4:**
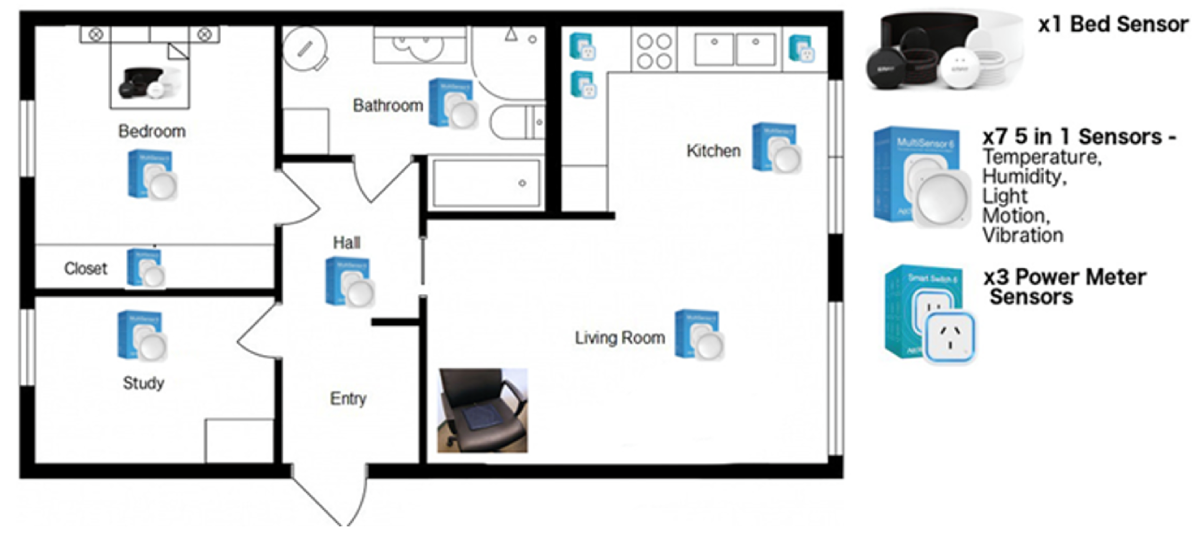
Passive sensors installed in the household to support independent living.

**Figure 5:**
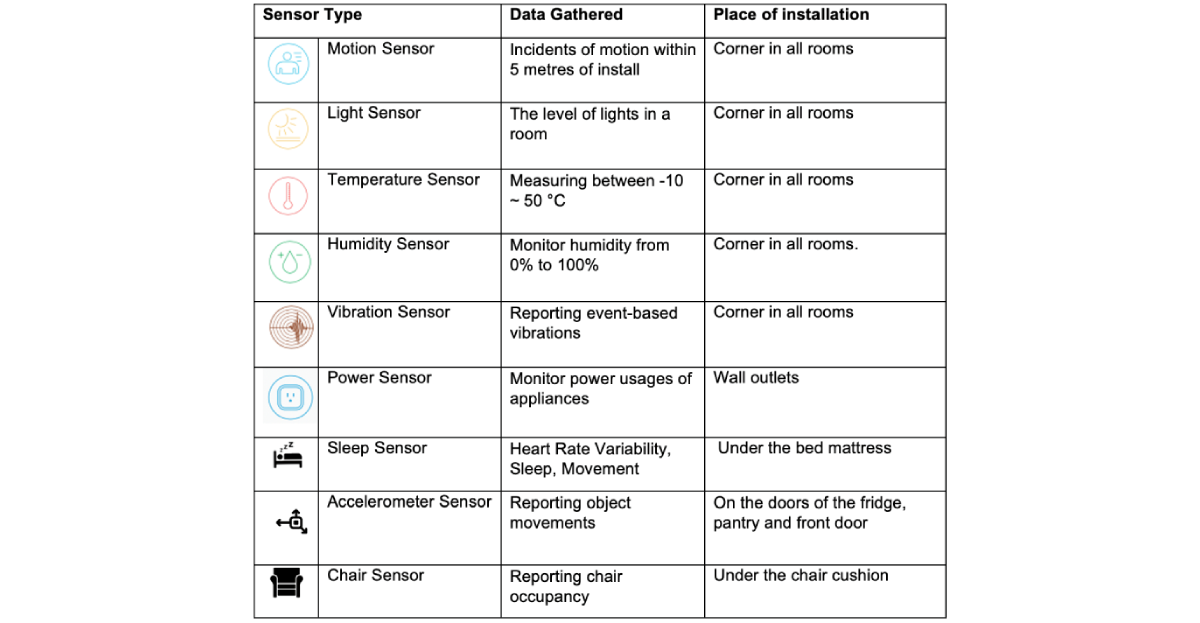
Description of Smart Safer Homes sensors deployed, the data gathered, and where these sensors were installed.

Figure 4 shows the spread of sensors installed in the household of the smart home group participants and Figure 5 provides a description of the SSH sensors deployed and where they were installed in the home.

**Table 3:** The mapping of sensors to daily living activity domains.

Daily living activities	Sensor type	Location
Indoor walking	Motion sensor	All rooms
Sit-stand transition times (out of a bed/chair)	Accelerometer, pressure sensor, sleep sensor	Bedroom, living room
Meal preparation	Motion sensor, electrical power sensor, accelerometers	Dining room, kitchen
Hygiene	Motion sensor, humidity sensor, temperature sensor	Bathroom
Dressing	Motion sensor, accelerometer	Wardrobe

#### Informal Carer(s)

Data on the burden experienced by the informal carers of the people participating in the trial were collected using the 4-item Zarit Screen (ZBI-12) survey [32] at all three time points (start, mid-trial, and end-trial). The ZBI-12 is a valid and reliable instrument for measuring the burden of carers. A total ZBI-12 score ranges from 0 to 48 based on the summation of 12 items; a score of 0-10 indicates no to a mild burden, 10-20 indicates a mild to moderate burden, and >20 indicates a high burden. The individual(s) identified as informal carer(s) by the participant were contacted by phone or email and the questionnaire was posted out or delivered through email (depending on preference).

#### Formal Carers: Aged Care Service Providers

The outcomes collected from the respective aged care service providers at the end of the trial are shown in Table 4.

**Table 4:** Data collected from aged care providers.

Outcome/objective	Data variable	Data source
Impact of care: case manager	Four-level self-completion questionnaire with eight attributes: control over daily life, personal cleanliness and comfort, food and drink, personal safety, social participation and involvement, occupation, accommodation cleanliness and comfort, dignity. Additional questions: subjective rating of health, open question	ACCOM^a^ measure (adapted from ASCOT^b^ for the Australian population)
Organizational change management and impact on workplace culture	Administrative/operational changes implemented/required to implement the SSH^c^ service	Semistructured interviews during focus groups with the formal carers
User perceptions of the SSH system	Ease of use, quality of training received, easy or hard to take and monitor clients’ measurement. Responsiveness of Project Officer to changes in O-ADLs^d^, effectiveness in improving ability to deliver care, impact on workload	Semistructured interviews during focus groups with the formal carers

^a^ACCOM: Australian Community Care Outcome Measurement.

^b^ASCOT: Adult Social Care Outcome Toolkit.

^c^SSH: Smarter Safer Homes.

^d^O-ADL: Objective Activities of Daily Living.

#### Government Data Source

The outcomes collected from the Commonwealth Government Department of Health (Services Australia) and the QLD Government Department of Health (hospital data custodians) at the end of the trial are shown in Table 5.

**Table 5:** Data collected at the administrator level.

Outcome/objective	Data variable	Data source
Hospitalization	Admitted hospital separations (same day or overnight) and emergency department presentations	Queensland Hospital admitted patient data collection (Queensland Health Statistical Services Branch), emergency department collection (Healthcare Improvement Unit)
Use of clinical services	Nonreferred (eg, general practitioner) attendances, attendances to specialists, allied health professionals, pathology (hematology, etc), diagnostic imaging (X-ray, etc)	Medicare benefits schedule claims, Services Australia
Pharmaceutical Benefits Scheme (PBS) expenditure	Prescription medications	PBS claims, Services Australia

### Data Management

Data were predominantly captured in the ethics-approved and secure REDCap system. All survey responses were collected through REDCap with the exception of interviews that were captured through recordings. Health services utilization data, already captured through business-as-usual processes of the relevant health services, were utilized in the analysis. Every participant was allocated a REDCap identifier. Once allocated, REDCap numbers were the only identifier of research participants. Interviews with aged care service providers were performed one-on-one with participants and therefore the interviewees were identifiable to the interviewer. 

### Statistical Methods

#### Data Collection and Missing Data

Survey results were collected at three time points: at the baseline, middle, and end of the study. The final survey data included in our analyses contain results from participants with a baseline survey and at least one follow-up and middle/end survey. For missing responses in surveys, we will conduct a case-by-case retrospective analysis with project officers and aged care service providers, and/or use multiple imputation methods to replace missing data.

#### Survey Data Analyses

At each survey time point, intergroup differences in survey results between the intervention and control groups will be analyzed by *χ*^2^ tests and Wilcoxon rank-sum tests. If significant differences in survey results are observed, a log-binomial regression model will be further used to estimate the relative risk of each group to changes of survey results. Within each group, for all three surveys, the Friedman nonparametric test will be used to understand intragroup differences in survey results. Similarly, if significant differences in survey results are observed, the Wilcoxon signed-rank test with Bonferroni correction will be used to further investigate pairwise differences between surveys.

#### Cost-Effective Analysis

A within-trial cost-utility analysis will be performed to assess the value for money of the intervention. The time frame will be the end of the trial, consistent with the trial, and the base case perspective will be from the government as the primary funder of health and social services. An incremental cost-effectiveness ratio will be calculated by collating the costs for the intervention and control groups, and using quality-adjusted life years (QALYs) gained as the outcome in the equation. QALYs will be derived using an area under the curve approach based on the health-related quality of life utility index values derived from responses to the EQ-5D-5L. Costs will comprise the costs of the intervention plus health resource use collected through routine databases (hospital, MBS, and PBS) with patient consent and community services support (eg, nursing, home care) collected during the trial period. Appropriate techniques to account for uncertainty in the estimates (eg, nonparametric bootstrapping) will be implemented. Costs will be summed and compared between groups, adjusting for baseline differences.

### Monitoring

#### Data Monitoring

Regular sensor data monitoring was undertaken by engineers in the team. Data checks occurred every 1-2 days. The organizational cybersecurity team was included to support the constant monitoring for cybersecurity threats throughout the data collection period. This monitoring included checking the functional integrity of the REDCap research database.

Any identified issues that required follow up were reported to the project manager for dissemination to the project steering group and/or the risk and safety committee. Due to the governance structures in place across expert engineering teams, and the two project governance committees, a separate data monitoring committee was not identified as a requirement.

#### Harms

A risk and safety committee was established from the commencement of the project. This committee consisted of an aged care service provider representative from each stakeholder partner, key project staff, and an expert geriatrician to provide any medical oversight required. This committee met bimonthly throughout the project and was available for assessing any unintended effects of the trial intervention or conduct.

#### Auditing

Two types of audits occurred throughout the trial. An audit of all documentation occurred at regular intervals. This was to ensure all consent forms were completed correctly and any errors could be addressed appropriately and in a timely manner.

Additionally, at the end of the installation phase, an audit was undertaken of sensor placements in the homes. This occurred to ensure consistency of sensor placements across homes.

### Ethics and Dissemination

#### Research Ethics Approval

Ethics approval was obtained from the Commonwealth Scientific and Industrial Research Organisation (CSIRO) Human and Medical Research Ethics Committee (CHMREC) on November 26, 2018 (Proposal # HREC 4/2018).

#### Protocol Amendments

After the original submission of ethics, which granted approval for the project to commence, there were amendments sought throughout the implementation stage. Amendments typically included additions and/or removal of researchers to the project and extension of approval dates.

#### Consent

Informed consent was obtained from all research participants, including the older adult participants, carers, and aged care service providers who were interviewed at the end of the trial. During the meeting, the project officer discussed the trial and its requirements with participants and their informal carer(s). Individuals who agreed to participate were asked to sign the ethics-approved Participant Information and Consent Form (see Multimedia Appendix 1). The participants consented that the information gained during this trial may be published and that they will not be identified, and their personal results will not be divulged. To collect MBS and PBS data, all participants were required to sign an additional, separate, Consent Form (according to Consent Trial Guidelines for researchers requesting access to MBS and PBS participant/provider information; see Multimedia Appendix 1). The wording in this form was reviewed by the Health Strategy Branch, Health Services Australia, and subsequently approved by the CHMREC.

#### Confidentiality

Personal information was collected through surveys approved by the ethics committee. Survey responses were collected via REDCap and did not contain names. Access to demographic information about participants that had potential to identify the participant was restricted. Those conducting the analysis were not provided with this information and only the project officers and engineers (who provided maintenance to the system) had access to individual information. These research professionals are experienced in dealing with sensitive information and they are aware of the implications of accessing this type of information unnecessarily and without reason.

#### Dissemination Policy

The trial results will be communicated through media release, conference presentations, journal publication, and a final report to all stakeholders involved in the research.

## Results

The aim was to recruit 200 participants and a total of 195 participants were finally recruited, with 97 randomized to the intervention group. The study also received participants’ clinical service data from government data resources in June 2021. Final data analysis is underway at present and final outcomes will be presented in a future publication.

## Discussion

### Study Significance

A crisis is looming with the increase in an aging population presenting a large burden on the health system and aged care services unable to support them. Digital technology solutions such as smart homes present real opportunities to address this crisis. Australian aged care reforms have focused on providing aged care support in older peoples’ homes. CSIRO has developed a co-designed digital solution, the SSH platform, which features a novel functional independence measure to monitor and support people living in their own home setting. To test this, an RCT was undertaken to evaluate the implementation of this platform in assisting aged care providers to provide timely care and support and improve the lives of older Australians in various geographical settings. The findings of this study will inform the benefits of digital solutions in the support of people aging in the community and defining new age care delivery pathways for more effective aged care in the home.

### Expected Findings

This RCT was designed to investigate whether the implementation of the SSH platform for independent-living older adults improves care service delivery, social and health-related quality of life, and reduces the carer burden and the cost to public health services.

### Strengths and Limitations

To the extent of our knowledge, this is the first large RCT to comprehensively assess the impact of a smart home–based technology on aged care service delivery, quality of life, and public health service costs. However, during the trial, the participants experienced several weeks of lockdowns in March, May, and August of 2020 due to the COVID-19 pandemic. Whether these unprecedented environmental changes had an impact on the study results is yet unknown.

## Appendix

Multimedia Appendix 1 Participant consent forms.

## Abbreviations

ACCOM: Australian Community Care Outcome Measurement
ADL: Activity of Daily Living
AMTS: Abbreviated Mental Test Score
ASCOT: Adult Social Care Outcome Toolkit
CHMREC: CSIRO Human and Medical Research Ethics Committee
CSIRO: Commonwealth Scientific and Industrial Research Organisation
iADL: Instrumental Activities of Daily Living
IoT: Internet of Things
MBS: Medicare Benefits Schedule
O-ADL: Objective Activities of Daily Living
PBS: Pharmaceutical Benefits Scheme
QALY: quality-adjusted life year
QLD: Queensland
RCT: randomized controlled trial
SSH: Smarter Safer Homes
ZBI: Zarit Burden Interview
