# Supplementary figures and images for: The Smarter Safer Homes Solution to Support Older People Living in Their Own Homes Through Enhanced Care Models: Protocol for a Stratified Randomized Controlled Trial
Source: JMIR Res Protoc. 2022 Jan 24;11(1):e31970. doi: 10.2196/31970 (PMC8822419; doi:10.2196/31970)

**The participant consent form of trial**


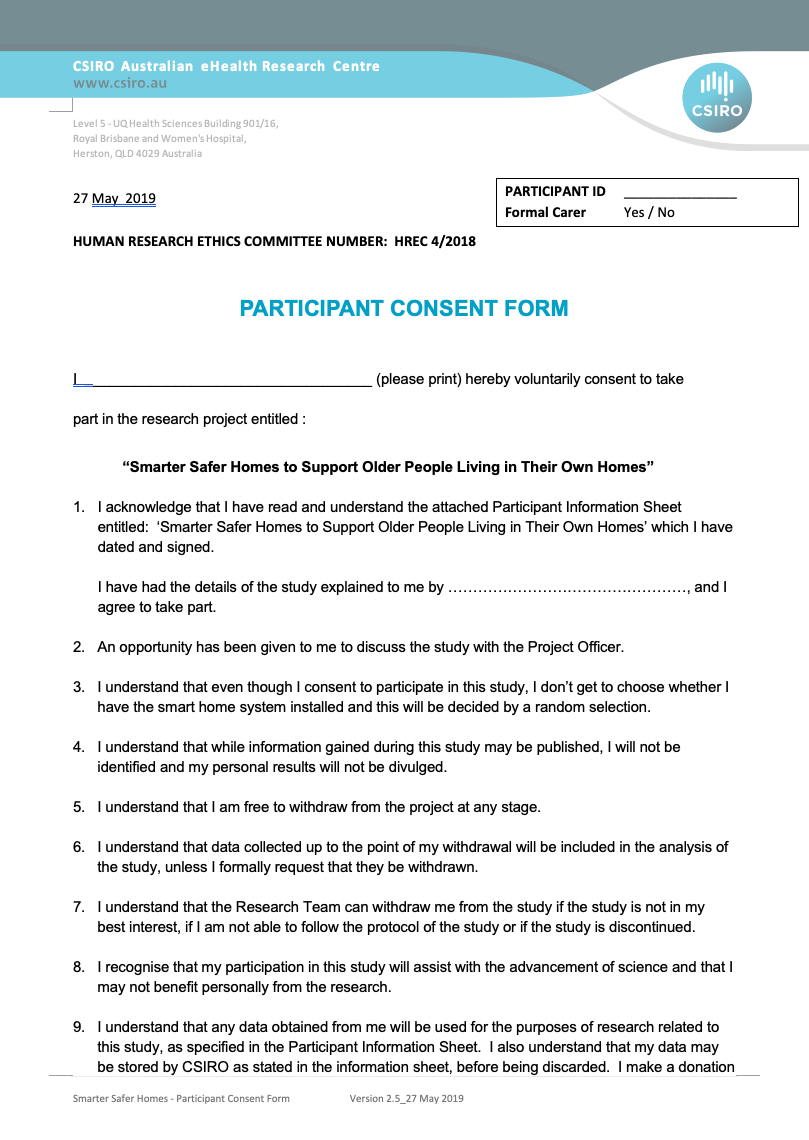


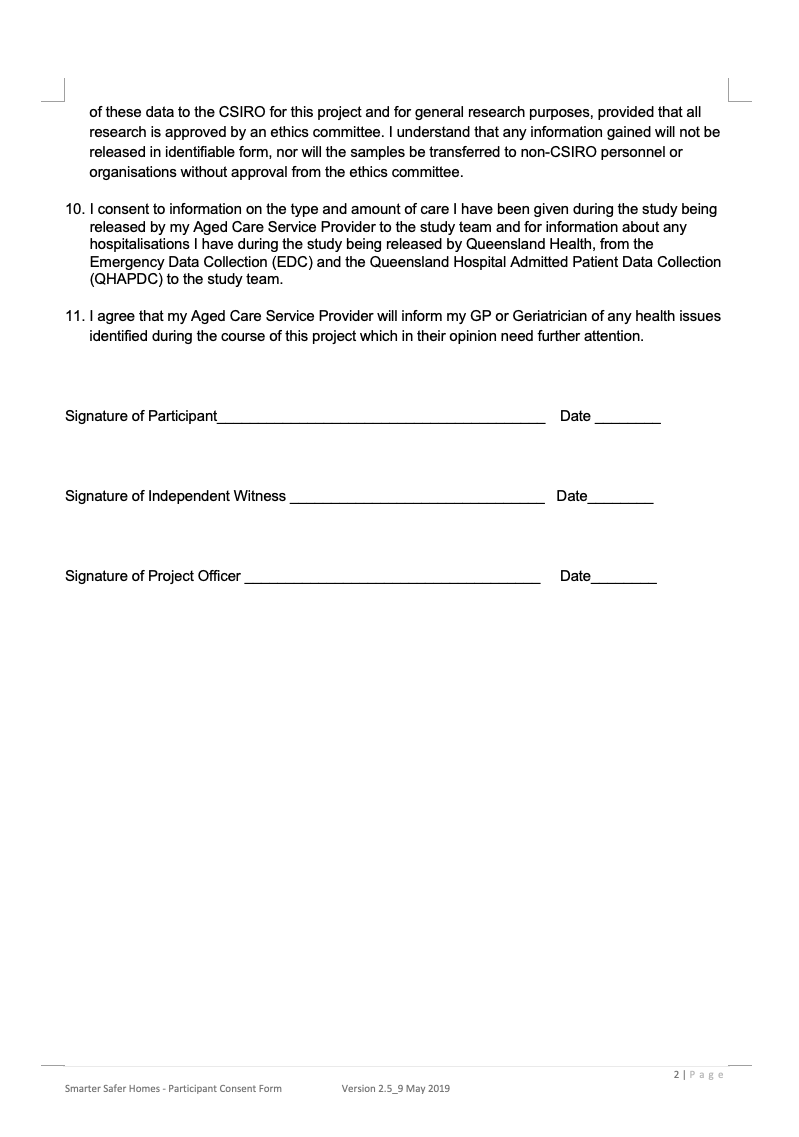


**The participant consent form of accessing MBS/PBS data**


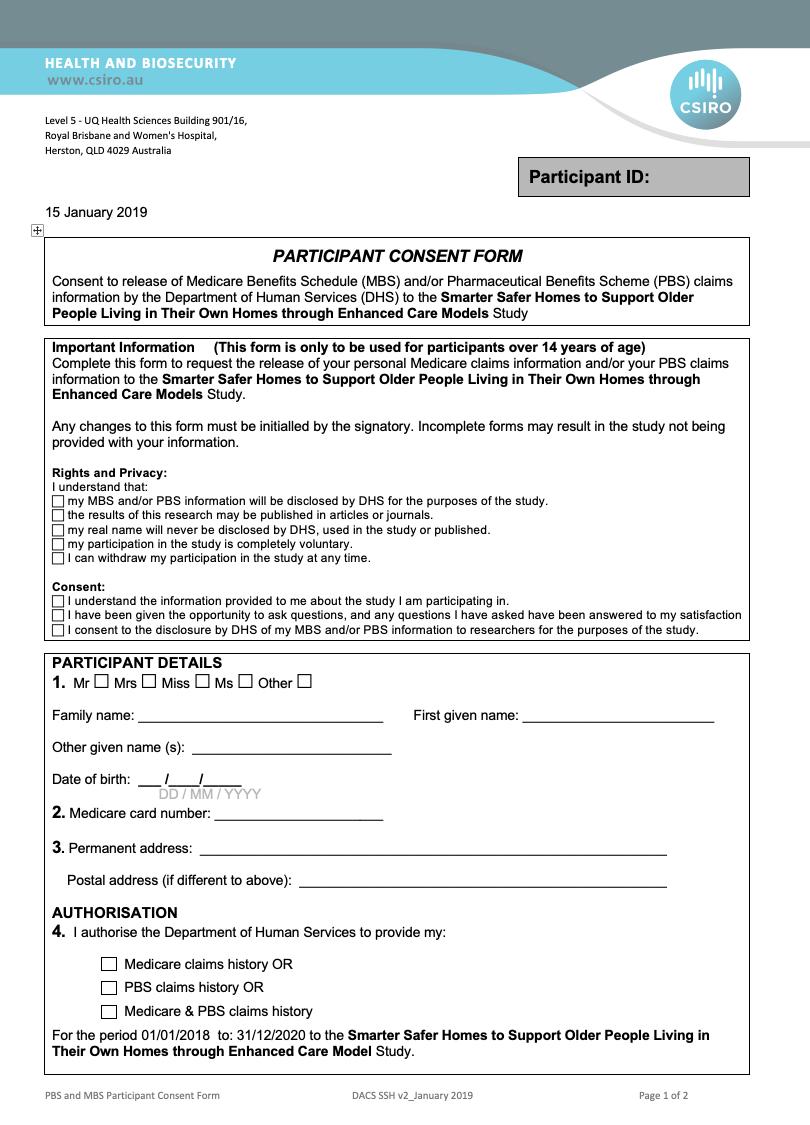


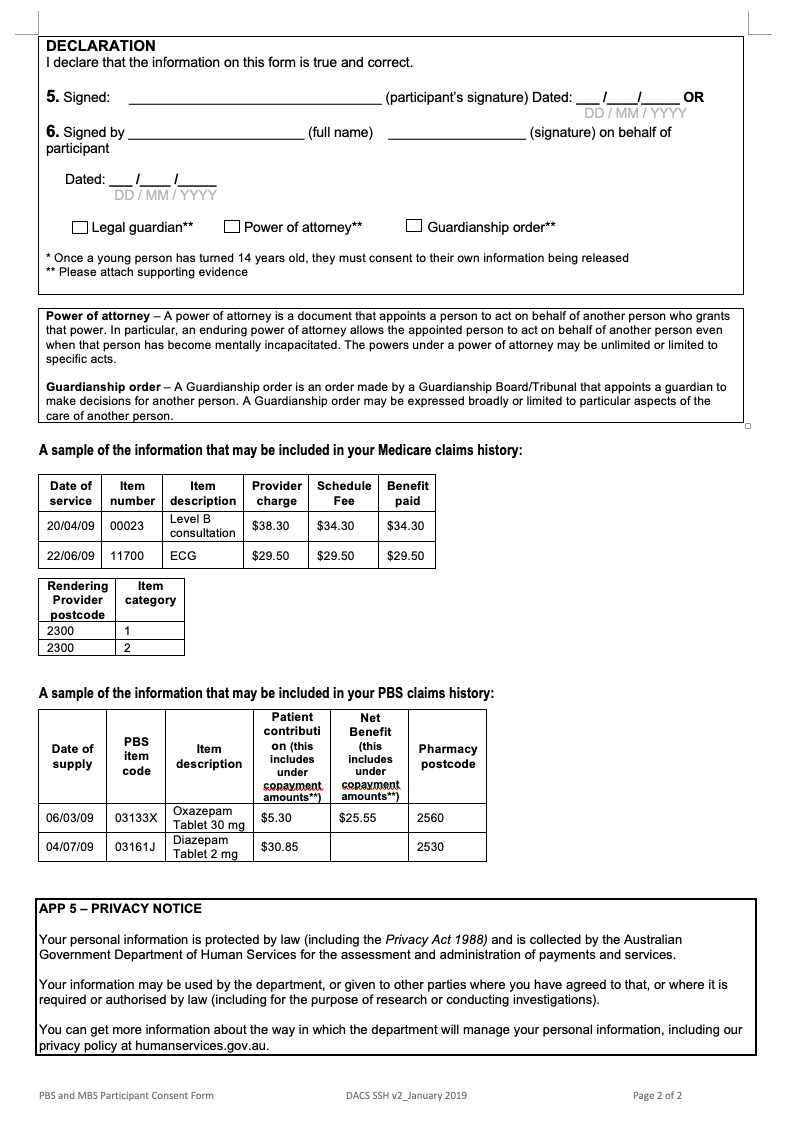

Supplement: Multimedia Appendix 1 [file resprot_v11i1e31970_app1.docx]
